# Identification of Potential Cerebrospinal Fluid Biomarkers To Discriminate between Infection and Sterile Inflammation in a Rat Model of Staphylococcus epidermidis Catheter Infection

**DOI:** 10.1128/IAI.00311-19

**Published:** 2019-08-21

**Authors:** Gwenn L. Skar, Matthew Beaver, Amy Aldrich, Dragana Lagundzin, Ishwor Thapa, Nicholas Woods, Hesham Ali, Jessica Snowden, Tammy Kielian

**Affiliations:** a Department of Pediatrics, University of Nebraska Medical Center, Omaha, Nebraska, USA; b Department of Pathology and Microbiology, University of Nebraska Medical Center, Omaha, Nebraska, USA; c Mass Spectrometry and Proteomics Core Facility, University of Nebraska Medical Center, Omaha, Nebraska, USA; d College of Information Science and Technology, University of Nebraska—Omaha, Omaha, Nebraska, USA; e Eppley Institute, University of Nebraska Medical Center, Omaha, Nebraska, USA; f Department of Pediatrics, University of Arkansas for Medical Sciences, Little Rock, Arkansas, USA; University of Illinois at Chicago

**Keywords:** biomarker, cerebrospinal fluid, cytokine, inflammation, proteome, shunt infection

## Abstract

Staphylococcus epidermidis cerebrospinal fluid (CSF) shunt infection is a common complication of hydrocephalus treatment, creating grave neurological consequences for patients, especially when diagnosis is delayed. The current method of diagnosis relies on microbiological culture; however, awaiting culture results may cause treatment delays, or culture may fail to identify infection altogether, so newer methods are needed. To investigate potential CSF biomarkers of S. epidermidis shunt infection, we developed a rat model allowing for serial CSF sampling. We found elevated levels of interleukin-10 (IL-10), IL-1β, chemokine ligand 2 (CCL2), and CCL3 in the CSF of animals implanted with S. epidermidis-infected catheters compared to sterile controls at day 1 postinfection. Along with increased chemokine and cytokine expression early in infection, neutrophil influx was significantly increased in the CSF of animals with infected catheters, suggesting that coupling leukocyte counts with inflammatory mediators may differentiate infection from sterile inflammation. Mass spectrometry analysis revealed that the CSF proteome in sterile animals was similar to that in infected animals at day 1; however, by day 5 postinfection, there was an increase in the number of differently expressed proteins in the CSF of infected compared to sterile groups. The expansion of the proteome at day 5 postinfection was interesting, as bacterial burdens began to decline by this point, yet the CSF proteome data indicated that the host response remained active, especially with regard to the complement cascade. Collectively, these results provide potential biomarkers to distinguish S. epidermidis infection from sterile postoperative inflammation.

## Abstract (precis)

Staphylococcus epidermidis cerebrospinal fluid (CSF) shunt infection is a common complication of hydrocephalus treatment, creating grave neurological consequences for patients, especially when diagnosis is delayed. The current method of diagnosis relies on microbiological culture; however, awaiting culture results may cause treatment delays, or culture may fail to identify infection altogether, so newer methods are needed.

## INTRODUCTION

Cerebrospinal fluid (CSF) shunt placement is the most common treatment for hydrocephalus, with up to 33,000 CSF shunts placed each year (1). Unfortunately, this life-saving procedure is frequently complicated by infection, with an incidence of 4% to 17% (2–5). Bacterial pathogens are responsible for the majority of shunt infections, with Staphylococcus epidermidis being the leading species (3). Bacterial cells adhere to the shunt tubing and form a biofilm, which is resistant to both immune-mediated clearance and antibiotics (6). Therefore, treatment for shunt infection requires both surgical removal of the shunt as well as long-term parenteral antimicrobial therapy, before a new shunt can be placed (1).

Currently, the diagnosis of shunt infection relies on isolating a pathogen from CSF culture; however, there are numerous challenges with this methodology (5, 7). First, conventional testing methods normally require 24 to 48 h to isolate organisms, and in the case of slow-growing organisms, several days may lapse before bacterial growth occurs (1, 3). Awaiting culture results may delay appropriate treatment, especially in the absence of other definitive laboratory results. Second, if a patient receives antibiotics prior to CSF collection, the culture may be falsely negative (8). Third, once a biofilm is formed, there may be very few detached or planktonic bacteria, which can also lead to falsely negative cultures (9).

In the case of a negative CSF culture, clinicians must rely on serum laboratory results, other CSF indices (i.e., white blood cell count [WBC], WBC differential, glucose, protein, and Gram stain), and clinical presentation to determine if an infection is present (7). However, laboratory results and clinical symptoms are neither sensitive nor specific for shunt infection (10). A study by Conen et al. demonstrated that patients with CSF shunt infection may have a normal CSF WBC, so clinicians cannot exclude the possibility of shunt infection with a normal WBC (11). Conversely, an elevated WBC does not necessarily correspond to infection. Several studies have described sterile postoperative meningitis characterized by an elevation of the CSF WBC after neurosurgical procedures, which can further complicate an infectious diagnosis (12–14). Therefore, new modalities are needed to swiftly and accurately diagnose CSF shunt infections to improve our ability to recognize and treat these infections. Early intervention may ameliorate the increased risk of intellectual disability, seizures, and lifelong morbidity and mortality associated with shunt infection (6).

The utility of CSF cytokines and chemokines as diagnostic markers for meningitis has been explored by several groups; however, data are extremely limited in the setting of CSF shunt infection (15, 16). Srinivasan et al. investigated the diagnostic utility of cytokines in neonates with meningitis, including a few patients with CSF shunts (10). This report revealed elevated levels of tumor necrosis factor alpha (TNF-α), interleukin-1 (IL-1), IL-6, IL-8, IL-10, and IL-12 in neonates with meningitis; however, none of these cytokines proved sufficient for diagnosis (10). A follow-up study by this group demonstrated that a combination of IL-23, IL-18, and soluble receptor for advanced glycation end product (sRAGE) levels was useful for diagnosing neonatal meningitis, including 4 patients with CSF shunts (17). Another report examined a limited number of CSF inflammatory markers associated with shunt infection, which found a correlation between higher vascular endothelial growth factor (VEGF) levels at the time of shunt placement and subsequent infection (18). However, this study examined cytokine levels not at the time of presentation of infection but rather at the time of initial shunt placement as a potential predictor of later infection (18). A report from our laboratory demonstrated that pediatric patients with Gram-positive shunt infection tended to have higher levels of the anti-inflammatory cytokine IL-10, while those with Gram-negative infection had higher levels of the proinflammatory mediators IL-1β, fractalkine (CX_3_CL_1_), chemokine ligand 2 (CCL2), and CCL3 (19).

In addition to the potential of cytokines and chemokines as biomarkers of CSF shunt infection, mass spectrometry (MS) can be used to identify proteins related to the host immune response and/or pathogen virulence factors that may prove useful from a diagnostic perspective. MS has been widely used to identify biomarker candidates in several neurodegenerative disorders, such as multiple sclerosis, Parkinson’s disease, and Alzheimer’s disease (20–22). Pertinent to CSF shunt infection, MS was used to identify soluble membrane attack complex (sMAC), a component of the complement system, to diagnose pyogenic shunt infection; unfortunately, it was not predictive for S. epidermidis, which accounts for >50% of all shunt infections (23). To expand CSF shunt infection diagnosis beyond its reliance on traditional bacterial culture, a multimodal approach will likely be needed. MS, in conjunction with leukocyte and chemokine/cytokine analysis, offers the possibility of profiling host biomarkers in the CSF to identify infection, despite frequent confounders such as contamination from skin flora, recent surgery, or pretreatment with antibiotics.

Here we utilized a rat model of S. epidermidis catheter infection that supports serial CSF sampling, to identify potential biomarkers of infection in the CSF that is routinely collected as part of clinical care in patients with suspected shunt infection. CSF samples from animals with S. epidermidis-infected catheters revealed elevated IL-10, IL-1β, CCL2, and CCL3 levels compared to sterile controls. Infected animals also displayed increased CSF neutrophil infiltrates at day 1 postinfection compared to controls, with a shift to predominately monocytes/macrophages at day 5 postinfection. MS analysis revealed an increased number of proteins in the CSF of infected animals, particularly related to the complement pathway. Interestingly, elevated expression levels of several proteins in the CSF persisted in the face of declining infectious burdens. These results have identified several potential biomarkers for further study in animal and human trials of S. epidermidis shunt infection.

## RESULTS

### Catheter-associated bacterial growth predominates in early infection, which is consistent with biofilm infection.

**FIG 1:**
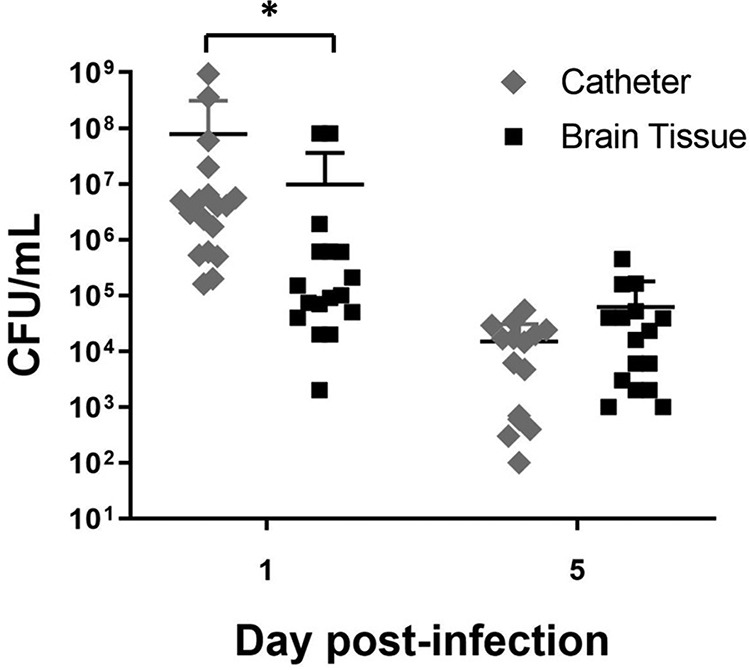
Kinetics of S. epidermidis growth on catheters versus surrounding brain tissue. Catheters were recovered from brain tissue at the indicated day postinfection and sonicated in PBS, whereupon the surrounding brain tissue was homogenized and bacterial titers were determined (*n* = 15 to 18). Catheter and brain tissue cultures from rats with sterile catheters were negative and are not presented (*, *P < *0.05 by Student’s *t* test).

To assess the retention of catheter-associated bacteria and examine the kinetics of infection, rats were implanted with catheters precoated with S. epidermidis 1457 bacteria. This approach has been widely used in mouse models of infection in the CSF and periphery (24, 25). At day 1 postinfection, catheters had a higher bacterial burden than surrounding brain tissues (*P = *0.001) (Fig. 1), which is consistent with biofilm growth as seen in a murine model of central nervous system (CNS) catheter infection (26). By day 5, bacterial burdens on catheters and in the surrounding brain tissue began to decline, with no statistically significant differences detected between the sites (*P = *0.149) (Fig. 1). Quantification of bacteria in the CSF was not performed due to limiting volumes, and animals receiving sterile catheters had no evidence of bacterial growth (data not shown).

### Cytokine and chemokine expression is enriched in the CSF during S. epidermidis catheter infection.

**FIG 2:**
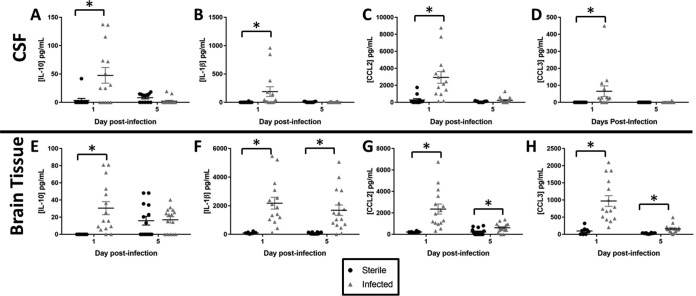
S. epidermidis catheter-associated infection elicits elevated cytokine/chemokine expression compared to sterile injury. Shown is quantitation of IL-10 (A and E), IL-1β (B and F), CCL2 (C and G), and CCL3 (D and H) in the CSF and brain tissue of rats with S. epidermidis-infected versus sterile catheters (*n* = 8 to 16) (*, *P < *0.05 by Student’s *t* test).

To assess the utility of CSF cytokines and chemokines as biomarkers for S. epidermidis catheter infection, CSF was analyzed for the presence of IL-1β, IL-6, IL-10, CCL2, CCL3, chemokine (C-X-C motif) ligand 1 (CXCL1), TNF-α, VEGF, and CX_3_CL_1_ by a multianalyte microbead array. Brain homogenates from tissues immediately surrounding the catheter tract were included for comparison. The CSF of animals with S. epidermidis-infected implanted catheters had significantly higher levels of IL-10 (*P = *0.005), IL-1β (*P = *0.002), CCL2 (*P* = <0.001), and CCL3 (*P* = <0.001) at day 1 postinfection than those of animals receiving sterile catheters (Fig. 2). Likewise, brain tissues of animals with S. epidermidis-infected catheters had higher levels of IL-10 (*P < *0.001), IL-1β (*P < *0.001), CCL2 (*P < *0.001), and CCL3 (*P < *0.001) at day 1 postinfection, demonstrating that the CSF inflammatory milieu mirrors that of the brain tissue during early infection. As bacterial burdens decreased at day 5 postinfection, levels of IL-1β (*P < *0.001), CCL2 (*P = *0.010), and CCL3 (*P < *0.001) remained significantly elevated in the brain tissue of animals with S. epidermidis-infected catheters (Fig. 2); however, the levels of these mediators were not elevated in the CSF (IL-1β, *P = *0.669; CCL2, *P = *0.112; CCL3, *P = *0.451) (Fig. 2). No significant differences in IL-10 levels were observed in the CSF (*P* = 0.054) or brain tissue (*P = *0.683) at day 5 postinfection (Fig. 2). In addition, no differences were noted in levels of IL-6, CXCL1, TNF-α, VEGF, or CX_3_CL_1_ in CSF or brain tissue at either time point (data not shown).

### CSF pleocytosis during S. epidermidis infection.

**FIG 3:**
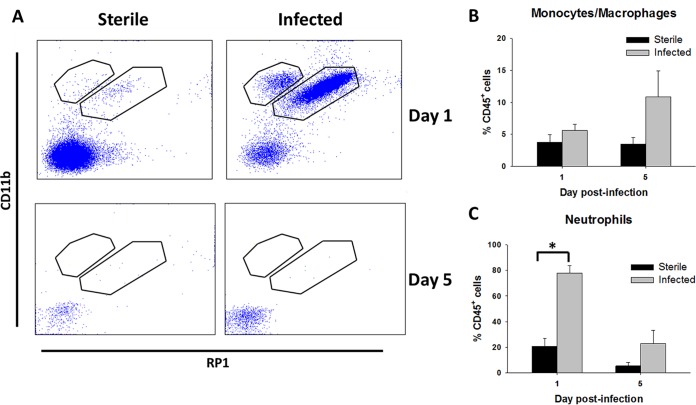
S. epidermidis catheter-associated infection elicits heightened leukocyte influx in the CSF compared to sterile postoperative inflammation. (A) Representative dot plots of CD11b and RP1 staining demonstrating CD45^+^ CD11b^+^ RP1^+^ neutrophils and CD45^+^ CD11b^+^ RP1^−^ monocytes/macrophages in the CSF of animals with S. epidermidis-infected versus sterile catheters at days 1 and 5 postinfection. (B and C) Percentages of CD45^+^ CD11b^+^ RP1^−^ monocytes/macrophages (B) and CD45^+^ CD11b^+^ RP1^+^ neutrophils (C) (*, *P < *0.05 by Student’s *t* test).

Since the levels of numerous chemokines were elevated in the CSF during S. epidermidis infection, we next examined whether this translated to changes in leukocyte influx. Neutrophil infiltrates (CD45^+^ CD11b^+^ RP1^+^) were significantly increased in the CSF of animals implanted with S. epidermidis-infected versus sterile catheters (*P = *0.001) (Fig. 3), consistent with human shunt infection (6). There were no significant differences in monocyte/macrophage influx (CD45^+^ CD11b^+^ RP1^−^) in the CSF of animals receiving infected versus sterile catheters at either time point (Fig. 3). The paucity of immunological reagents in the rat precluded us from discriminating monocytes from macrophages. The reduction in bacterial burdens from days 1 to 5 postinfection was associated with a shift from a predominantly neutrophil to a monocyte/macrophage infiltrate in the CSF.

### The host CSF proteome differs in S. epidermidis infection compared to sterile postoperative inflammation.

**FIG 4:**
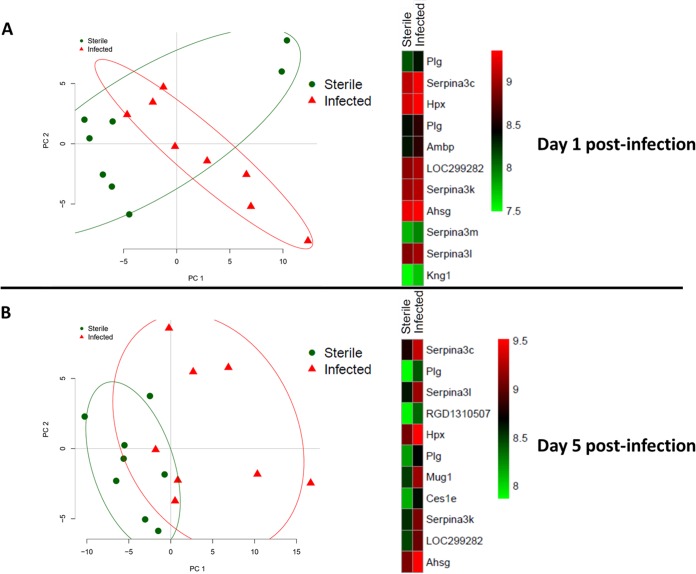
Principal-component analysis and heat map of significantly differentially expressed proteins in animals with S. epidermidis-infected versus sterile catheters at day 1 (A) and day 5 (B) postimplantation. Protein expression data were filtered for values that were present in both sterile and infected groups at days 1 and 5 postimplantation. Principal-component analysis was performed on this multidimensional expression profile to reduce the dimension to fewer components. Heat maps of expression for some of the highly varying proteins were created. Plg, plasminogen; Serpina3c, RCG20603; Hpx, hemopexin; Ambp, protein AMBP; LOC299282, serine protease inhibitor; Serpina3k, serine protease inhibitor A3K; Ahsg, alpha-2-HS glycoprotein; Serpina3m, serine protease inhibitor A3M; Serpina3l, serine protease inhibitor A3L; Kng1, kininogen 1; RGD1310507, similar to RIKEN cDNA 1300017J02; Mug1, murinoglobulin-1; Ces1e, carboxylesterase 1E.

Principal-component analysis (PCA) revealed distinctions between the CSF proteomes of sterile and infected animals at both days 1 and 5 postimplantation (Fig. 4). Heat maps of protein expression in the CSF at day 1 showed similar patterns between animals with sterile and S. epidermidis-infected catheters (Fig. 4). At day 5, protein expression levels in the CSF of sterile and infected animals diverged, with values in the sterile group remaining similar to those on day 1 postimplantation (Fig. 4).

**FIG 5:**
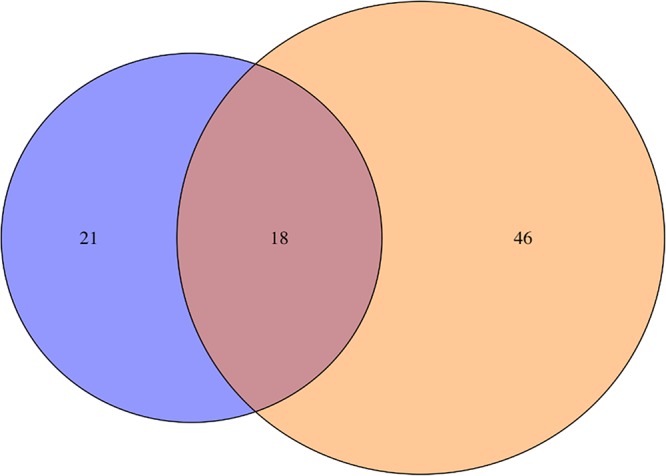
Relationship between differentially expressed proteins at days 1 and 5 in the CSF of S. epidermidis-infected animals. A Venn diagram depicts the similarities and differences between significantly differentially expressed proteins in the CSF of S. epidermidis-infected animals as determined by mass spectrometry analysis. Significant proteins in each category are presented in Tables 1 to 3.

MS data were also evaluated using differential expression analysis. At day 1, a total of 39 proteins were differentially expressed between sterile and infected groups, including several components of the complement cascade that were significantly enriched during infection (Fig. 5 and Table 1). The number of differentially expressed proteins was dramatically increased at day 5 postinfection, with a total of 64 (Fig. 5 and Table 2), again including numerous complement components. Eighteen proteins were differentially expressed in infected catheters at both time points (Fig. 5 and Table 3), two of which were complement components.

**TABLE 1:** Significant proteins in the CSF at day 1 postinfection by differential expression analysis

UniProt accession no.	Protein	Fold change	*P* value
A0A0G2K3K2	Actin, cytoplasmic 1	18.914	<0.001
A1Z0K8	Beta-actin (fragment)	14.606	<0.001
Q7TQ70	Ac1873	8.2054	<0.001
A0A0U1RRP9	Complement factor B	5.8239	<0.001
B5DEH7	C1r protein	5.4347	<0.001
F1M8E9	Lysozyme	4.976	0.001
A0A0G2K9I6	Ceruloplasmin	2.8446	0.001
G3V7L3	Complement C1s subcomponent	2.2829	<0.001
Q99PS8	Histidine-rich glycoprotein	2.2467	<0.001
A0A096MKF9	Complement factor B (fragment)	2.2298	<0.001
D4A1S0	Complement C1s subcomponent	1.9401	<0.001
P26644	Beta-2-glycoprotein 1	1.938	0.001
F1M8F5	Serine protease inhibitor A3F-like	1.8894	0.002
P36953	Afamin	1.7853	0.007
Q5FX35	Alpha 2 macroglobulin cardiac isoform (fragment)	1.6273	0.016
P09005	Serine protease inhibitor 2.1 (fragment)	1.6211	0.011
Q64599	Hemiferrin	1.513	0.009
P04916	Retinol-binding protein 4	0.6024	0.003
F1LNY3	Neural cell adhesion molecule 1	0.5884	0.012
D3ZVB7	Osteoglycin	0.5472	0.001
Q80ZA3	Alpha-2 antiplasmin	0.5437	<0.001

**TABLE 2:** Significant proteins in the CSF at day 5 postinfection by differential expression analysis

UniProt accession no.	Protein	Fold change	*P* value
B2RYM3	Inter-alpha trypsin inhibitor, heavy chain 1	4.6744	<0.001
Q63041	Alpha-1-macroglobulin	4.59087	<0.001
P04639	Apolipoprotein A-I	4.08858	<0.001
P08932	T-kininogen 2	3.98476	<0.001
Q64240	Protein AMBP	3.77439	<0.001
Q03626	Murinoglobulin-1	3.73376	<0.001
P02091	Hemoglobin subunit beta-1	3.55158	0.003
P14046	Alpha-1-inhibitor 3	3.53142	<0.001
Q68FT8	RCG33981, isoform CRA_a	3.45464	<0.001
Q63108	Carboxylesterase 1E	3.18616	<0.001
P10959	Carboxylesterase 1C	3.15715	<0.001
Q6TXE2	LRRGT00057	3.11604	<0.001
Q7TP05	Complement factor B	3.08502	<0.001
P01026	Complement C3	2.94677	<0.001
M0RBF1	Complement C3	2.89052	<0.001
Q6MG74	B-factor, properdin	2.81297	<0.001
A0A0G2K9Y5	Histidine-rich glycoprotein	2.79903	<0.001
P02770	Serum albumin	2.7432	0.005
A0A0G2JVX7	Apolipoprotein A-IV	2.64379	<0.001
P02651	Apolipoprotein A-IV	2.6223	<0.001
P20059	Hemopexin	2.611	<0.001
Q68FY4	Group-specific component	2.59445	<0.001
P05544	Serine protease inhibitor A3L	2.56083	<0.001
Q01177	Plasminogen	2.52346	<0.001
A0A0G2JSK1	RCG20603	2.50962	<0.001
F1LM05	Serine protease inhibitor	2.45796	<0.001
A0A0G2K1T8	Serine protease inhibitor	2.44569	<0.001
P05545	Serine protease inhibitor A3K	2.44023	0.001
P24090	Alpha-2-HS glycoprotein	2.30242	<0.001
A0A0G2K509	Keratin, type II cytoskeletal 5	2.25006	0.001
Q63556	Serine protease inhibitor A3M (fragment)	2.2289	<0.001
F1LR92	Serine protease inhibitor A3M	2.22372	0.001
P17475	Alpha-1-antiproteinase	2.12764	<0.001
B0BNN3	Carbonic anhydrase 1	2.10476	0.029
Q6TUG7	LRRGT00077	2.09422	<0.001
B5DF65	Biliverdin reductase B	2.04193	0.033
Q6AY07	Fructose-bisphosphate aldolase	1.88852	0.006
P05065	Fructose-bisphosphate aldolase A	1.88407	0.013
P02680	Fibrinogen gamma chain	1.87964	0.036
Q7TP75	Aa2-066	1.84315	<0.001
Q9Z1G8	Fibronectin (fragment)	1.73653	0.003
P31720	Complement C1q subcomponent subunit A	1.67047	0.001
F1LST1	Fibronectin	1.43986	0.025
D3ZZR3	Cathepsin S	0.68612	0.028
P01015	Angiotensinogen	0.66514	0.026
P14841	Cystatin-C	0.60479	0.002

**TABLE 3:** Significant proteins detected in the CSF at both days 1 and 5 postinfection by differential expression analysis

UniProt accession no.	Protein	Day 1 fold change	Day 1 *P* value	Day 5 fold change	Day 5 *P* value
Q4G044	Fga protein (fragment)	2.9676	<0.001	2.56801	<0.001
A0A0G2KAY3	Kininogen 1	2.0586	0.011	3.91449	<0.001
P01048	T-kininogen 1	2.0181	0.013	3.55836	<0.001
Q5PQU1	Kininogen 1	1.9398	0.014	3.31211	<0.001
Q5XJW6	Cfh protein	1.9208	0.001	2.46363	<0.001
Q6QI47	LRRGT00161	1.8929	0.002	2.77448	<0.001
Q3T940	Apolipoprotein H (fragment)	1.891	0.001	2.74592	<0.001
Q5I0M1	Apolipoprotein H	1.8303	0.007	2.72986	<0.001
A0A0H2UHM3	Haptoglobin	1.824	0.005	3.12776	<0.001
Q6P734	Plasma protease C1 inhibitor	1.7339	0.001	1.618	0.009
Q4KM66	LOC500183 protein	1.7302	0.018	2.64542	<0.001
Q7TP84	Plasminogen	1.7265	0.004	3.16419	<0.001
A0A0G2K896	Similar to RIKEN cDNA 1300017J02	1.6411	0.004	2.4633	<0.001
Q9QX79	Fetuin-B	1.6111	0.016	2.58171	<0.001
P12346	Serotransferrin	1.4392	0.009	1.78636	<0.001
P31721	Complement C1q subcomponent subunit B	1.3483	0.014	1.8446	<0.001
P02767	Transthyretin	0.6425	<0.001	0.74893	0.036
P22057	Prostaglandin-H2 D-isomerase	0.6121	0.01	0.39406	<0.001

Pathway analysis of the differentially expressed proteins identified several biological processes that were active during S. epidermidis catheter infection (see Table S1 in the supplemental material). At day 1 postinfection, a total of 6 biological processes were enriched, with 2 to 7 proteins present in each category. At day 5 postinfection, the number of biological processes increased to 13, even in the context of reduced bacterial burdens. When comparing pathways that were common between the two time points, the number of proteins at day 5 postinfection was increased, suggesting progressive pathology.

**FIG 6:**
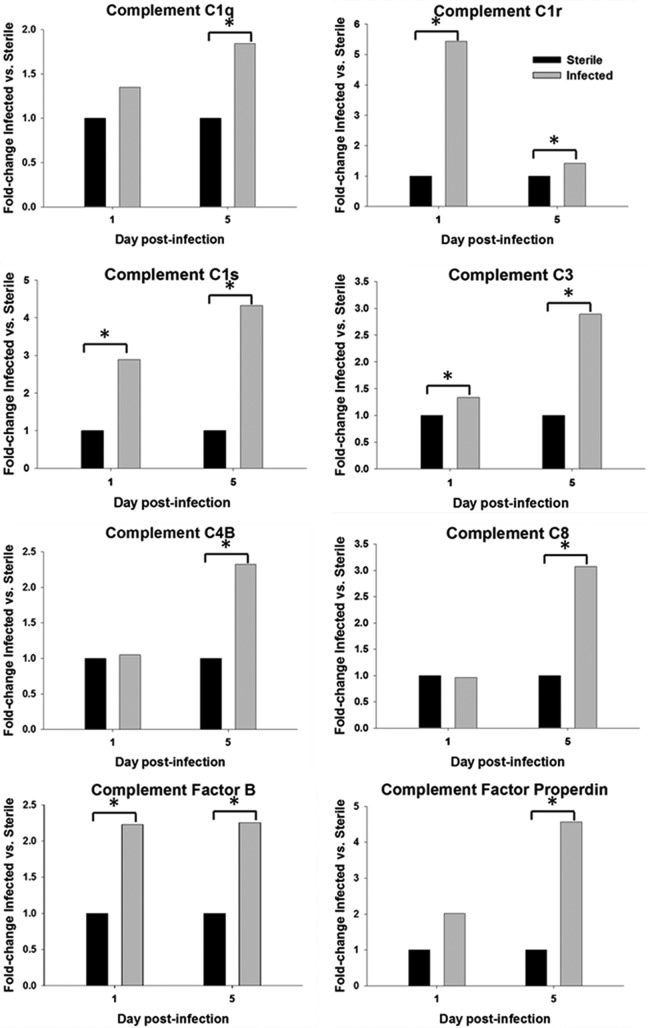
Levels of proteins associated with the complement pathway are significantly elevated in the CSF of animals with S. epidermidis-infected versus sterile catheters. The abundances of various complement components in the CSF were determined by mass spectrometry and are presented as fold changes for S. epidermidis-infected versus sterile animals (*, *P < *0.05 by a Wilcoxon signed-rank test).

As alluded to above, MS analysis revealed that several components of the complement cascade were differentially expressed. A subanalysis of all detected complement proteins was performed using a Wilcoxon signed-rank test. At day 5 postinfection, two fragments of complement C1q were significantly different between sterile and infected groups (UniProt accession numbers P31720 [*P = *0.005] and P31721 [*P = *0.024]) (Fig. 6 and data not shown). Additional complement components that were significantly enriched in the CSF at day 5 postinfection included complement C4B (*P = *0.003), complement C8 chains (UniProt accession numbers D3ZWD6 [*P = *0.048] and D3ZPI8 [*P = *0.004]), complement factor properdin (*P = *0.005), and complement C3 (UniProt accession number P01026 [*P = *0.009]) (Fig. 6 and data not shown).

The levels of two complement C1s fragments (UniProt accession numbers D4A1S0 [*P = *0.013 and *P* = 0.001] and Q6P6T1 [*P = *0.005 and *P = *0.034]), complement C1r (*P < *0.001 and *P* = 0.006), complement C3 (UniProt accession number MORBF1 [*P = *0.010 and *P = *0.009]), and complement factor B fragment (*P = *0.031 and *P = *0.011) were significantly higher in the infected group at both days 1 and 5 (Fig. 6 and data not shown). Importantly, the levels of all complement proteins were elevated at day 5 postinfection, a time point when bacterial burdens were declining, suggesting that their expression is driven by other signals.

## DISCUSSION

CSF shunt infection creates a significant burden on the health care system and families, due to both acute-care needs and long-term neurological consequences, such as decreased IQ and seizures (4, 27–29). Currently, the diagnosis of these infections relies on bacterial cultures, as other CSF indices are not sensitive or specific (10). However, cultures may be falsely negative due to the difficulty of detecting biofilm-associated bacteria prior to device removal, the prevalence of slow-growing organisms associated with shunt infection, and frequent treatment of patients with antibiotics prior to CSF collection. While detection of bacterial proteins or DNA in the CSF may be useful, S. epidermidis and Propionibacterium acnes are key pathogens in this setting as well as normal skin flora for humans (1, 3, 7). The presence of their proteins or DNA in CSF may represent contamination, so it is essential to determine host biomarkers that are elaborated in response to infection (30). More sensitive and specific modalities are needed for the diagnosis of shunt infection to ensure that patients receive necessary treatment in a timely manner.

To explore biomarker discovery in shunt infection, we developed the first rat model of CNS catheter infection. We elected to use S. epidermidis as the infecting organism in our model as this organism causes two-thirds of all human shunt infections (3). This model allows for serial CSF sampling in the same animal, which is an extremely valuable tool for longitudinal analysis and tracking of CSF biomarkers. Findings in this model can subsequently be explored and validated as clinical diagnostic markers for human CSF shunt infection. We demonstrate that the S. epidermidis bacterial burden is significantly higher on the catheter than in surrounding brain tissue during acute infection, which is consistent with a murine model of catheter-associated biofilm infection, supporting its relevance as an *in vivo* model of human shunt infection (26). This is further supported by our CSF findings demonstrating a distinct CSF phenotype in infected versus sterile catheter placement, similar to human infection (6, 19).

Studies of pediatric meningitis, some of which have included a small number of patients with CSF shunt infection, have reported elevated levels of chemokines and cytokines in the CSF (15, 16, 31–35). In our rat S. epidermidis catheter infection model, the levels of several mediators, including IL-10, IL-1β, CCL2, and CCL3, were significantly increased in both the CSF and brain tissue at day 1 postinfection. The elevation of CSF IL-10 levels is consistent with data from our previous human study demonstrating increased levels of IL-10 in patients with Gram-positive shunt infections and the key role for IL-10 in regulating the inflammatory response in a murine model of S. epidermidis CNS catheter infection (19, 36). At day 1 postinfection, elevated IL-1β, CCL2, and CCL3 expression in the CSF mirrored that in the brain parenchyma. However, although levels of these mediators remained elevated in brain tissue at day 5 postinfection, they were reduced in the CSF, which coincided with a decrease in bacterial burdens. Therefore, IL-1β, CCL2, and CCL3 may be useful biomarkers for tracking infection and determining when antibiotic treatment could be terminated as well as for diagnosis, although additional studies are needed to validate this possibility.

In the absence of a positive bacterial culture, the CSF WBC is frequently used clinically as a marker of infection in humans; however, some of the neurosurgical literature suggests that this represents sterile postoperative meningitis following CSF shunt placement (12–14). Consistent with these reports, our study demonstrated a modest neutrophil influx into the CSF in response to sterile catheter placement. However, neutrophil recruitment in the CSF was significantly elevated in animals with S. epidermidis-infected versus sterile catheters at day 1 postinfection. Therefore, the combination of significant pleocytosis with elevated levels of inflammatory mediators, such as IL-1β, CCL2, and CCL3, may differentiate modest procedure-related sterile meningitis from an active infection. Unlike neutrophils, differences in monocyte/macrophage infiltrates between infected and sterile groups were not as robust. The similarities in monocytes/macrophages at day 1 postinfection may result from resident choroid plexus macrophages, while the trend toward elevated levels of monocytes/macrophages seen at day 5 in S. epidermidis-infected versus sterile catheters could represent a wound-healing response, although these possibilities remain speculative. While our rat model demonstrates a shift from neutrophil to monocyte/macrophage infiltrates over the course of infection, these findings may not be translatable to humans, as it is difficult to know when infection is established in relation to when CSF is sampled in the clinical setting.

MS analysis with subsequent functional enrichment identified differences in the CSF proteomes between sterile and infected animals as well as temporal changes in protein abundance. The CSF proteome in sterile animals was similar to that in S. epidermidis-infected animals at day 1; however, by day 5 postinfection, there was an expansion in the number of differently expressed proteins in the CSF compared to the sterile group. This was intriguing, since bacterial burdens began to decrease at day 5 postinfection, yet the CSF proteome data indicated that the host response remained active. This is corroborated by the increase in the numbers of significantly enriched biological pathways and associated proteins in each category. Future studies will explore the kinetics of infection resolution as well as the longevity of elevated CSF protein levels. It is possible that the increase in protein expression in the CSF at day 5 postinfection is secondary to acute tissue damage that occurs at earlier intervals, which remains an open question.

An intriguing finding in this study was the identification of numerous complement proteins that were significantly increased in the CSF and not driven by bacterial burdens, since they had declined by this interval (i.e., day 5 postinfection). Complement proteins have been found to play significant roles in synaptic pruning during CNS development as well as in a variety of neurological disorders, such as Alzheimer’s disease and multiple sclerosis (37–43). Given the increase in CSF complement proteins in the context of reduced infection, we propose that complement may contribute to the deleterious neurological sequelae associated with CSF shunt infections. Ongoing studies in our laboratory are exploring the link between complement and neurological damage in shunt infection.

There are several limitations to this study. First, as mentioned above, immunological reagents for the rat are limited, which restricted our ability to discriminate between monocytes and macrophages in the CSF; nevertheless, our results are consistent with those from human studies of shunt infection and meningitis (5, 10, 12–15, 17). Second, as we mention above, our results may not be immediately translatable to those of human studies, as it is difficult to know when human infection is established compared to when CSF is obtained in a clinical setting. This demonstrates the importance of animal models of infection, to identify the initial signals of infection and opportunities for early intervention. Finally, while S. epidermidis is the most common cause of CSF shunt infections, other bacteria are capable of causing disease. Our previous pilot study revealed that in human CSF, there were differences in CSF inflammatory mediator concentrations in Gram-positive versus Gram-negative infections, with higher levels of the proinflammatory cytokines IL-1β, CX_3_CL_1_, CCL2, and CCL3 seen in response to Gram-negative shunt infection (19). To the best of our knowledge, there are no other studies that have looked at biomarkers in response to Gram-negative or fungal CSF shunt infection; however, it is very likely that host biomarkers will vary based on the type of infecting organism (33). To address this, our laboratory is currently using this rat model with other bacterial species to determine biomarkers that will be relevant to multiple pathogens.

We have created a unique rat model of S. epidermidis catheter infection that allows for serial CSF sampling, mimicking human shunt infection. This model is a powerful tool that can be used to identify novel CSF markers for diagnosis and potential monitoring of shunt infection. We found increased levels of IL-1β, CCL2, and CCL3 concomitant with elevated neutrophil infiltrates in the CSF of animals with S. epidermidis-infected catheters, which may serve as potential markers for distinguishing infection from sterile postoperative inflammation in humans. Proteome studies of the CSF revealed many complement proteins with elevated levels at day 5 postinfection, when bacterial burdens were declining. Studies are ongoing in our laboratory to further investigate complement components as markers for infection as well as their potential role in neurological consequences associated with shunt infections.

## MATERIALS AND METHODS

### Animals.

All experiments were performed using equal numbers of 8-week-old male and female Lewis rats (Charles River Laboratories, Wilmington, MA). The protocol for animal use was approved by the University of Nebraska Medical Center Institutional Animal Care and Use Committee (protocol number 16-091-09-FC) and is compliant with National Institutes of Health guidelines for the use of rodents. Animals were housed 2 to 3 per cage with species-appropriate enrichment in a 12-h light-dark cycle. Food and water were available *ad libitum*. Animals had a 3-day acclimation period after shipment prior to performance of any procedures. Group sizes were as follows: 8 to 12 per group for chemokine/cytokine analysis, 4 to 5 per group for flow cytometry analysis, and 8 to 9 per group for mass spectrometry analysis.

### Bacterial strain.

S. epidermidis 1457 was kindly provided by Paul Fey (University of Nebraska Medical Center). This isolate was recovered from a central venous catheter and characterized by Mack et al. and was previously used in a murine model of central nervous system catheter infection (24, 44). This isolate has not been laboratory adapted or modified since its characterization.

### Catheter preparation and implantation.

Hollow silicone catheters (4 mm long and 1 mm in diameter; Cook Medical Inc., Bloomington, IN) were incubated overnight with S. epidermidis 1457 to ensure bacterial adherence to the catheter and prevent bacterial efflux as previously described, which resulted in reproducible catheter colonization of 1.25 × 10^8^ CFU/catheter (24). Rats were anesthetized with an intraperitoneal (i.p.) injection of ketamine and xylazine (87 mg/kg and 13 mg/kg of body weight, respectively). To mitigate potential pain, animals received a subcutaneous (s.c.) injection of bupivacaine (1 mg/kg) and buprenorphine (0.01 mg/kg). The head was shaved, and a 1-cm longitudinal incision was made in the scalp. The rat was positioned in a stereotactic apparatus, and a burr hole was made in the skull using a 16-gauge needle at the following coordinates to direct catheter placement into the left lateral ventricle of the brain: −1.0 mm anterior-posterior (AP), 2.4 mm medial-lateral (ML), and 4.0 mm dorsal-ventral (DV) (45). To secure the catheter into place and prevent bacterial efflux or bleeding, the burr hole was sealed with bone wax. Vetbond surgical glue was used to close the scalp incision (3M, St. Paul, MN).

### Terminal CSF collection for cytokine/chemokine analysis.

At days 1 and 5 after catheter insertion, rats were anesthetized with an i.p. injection of ketamine and xylazine (87 mg/kg and 13 mg/kg, respectively), the back of the neck was shaved, and animals were placed in a stereotactic apparatus. Once positioned, the back of the neck was swabbed with betadine, and a percutaneous cisterna magna puncture was performed using a 25-gauge winged infusion set (Terumo Corporation, North America) with a guard to leave 4 mm of the needle tip exposed (46). A total of 100 to 120 μl of CSF was withdrawn and stored at −80°C until analysis.

### Bacterial enumeration from catheters and brain parenchyma.

Catheters and brain tissue were collected at days 1 and 5 postinfection as previously described (24, 26). Briefly, catheters were sonicated for 5 min in 500 μl of phosphate-buffered saline (PBS). The surrounding tissue within 2 mm of the catheter tract was homogenized in 500 μl of sterile PBS supplemented with a complete protease inhibitor cocktail tablet (Roche, Basel, Switzerland) and an RNase inhibitor (Promega, Madison, WI) using a Polytron homogenizer (Brickmann Instruments, Westbury, NY). A 100-μl aliquot of brain homogenates and the supernatant from sonicated catheters were used to quantitate bacterial titers by 10-fold serial dilution on tryptic soy agar plates. The brain tissue homogenates were also used to determine cytokine/chemokine levels by Milliplex analysis as described below (24).

### Cytokine/chemokine analysis.

Levels of inflammatory mediators in the CSF and brain tissue were measured with a rat microbead array system according to the manufacturer’s instructions (Milliplex; EMD Millipore Corp., Billerica, MA). This assay allowed for simultaneous measurement of 9 different cytokines and chemokines in a single 50-μl CSF or brain supernatant sample, including IL-1β, IL-6, IL-10, CCL2, CCL3, chemokine (C-X-C motif) ligand 1 (CXCL1), TNF-α, vascular endothelial growth factor (VEGF), and fractalkine (CX_3_CL_1_). Results were analyzed using Multiplex Assay Analysis software and normalized to the sample volume.

### CSF flow cytometry.

To characterize CSF leukocytes associated with S. epidermidis catheter infection, CSF was collected as described above. Immune cell populations were identified by fluorescence-activated cell sorter (FACS) analysis based on the expression of characteristic cell markers: macrophages/monocytes (CD45^+^ CD11b^+^ RP1^−^) and neutrophils (CD45^+^ CD11b^+^ RP1^+^) (47, 48).

### CSF collection for label-free mass spectrometry.

For serial collection of CSF at days 1 and 5 postinfection, rats were anesthetized with inhaled isoflurane (1% to 5% to effect). The back of the neck was shaved, and animals were placed in a stereotactic apparatus. Once positioned, the back of the neck was swabbed with betadine, and a percutaneous cisterna magna puncture was performed using a 25-gauge winged infusion set with a guard to leave 4 mm of the needle tip exposed (Terumo Corporation, North America) (46). A total of 100 to 120 μl of CSF was collected and stored at −80°C. Animals then received 500 μl of sterile PBS i.p. to prevent dehydration and replenish CSF.

### Label-free mass spectrometry.

CSF samples were processed by electrophoretic protein separation. Gels were then stained with Coomassie brilliant blue G-250 dye (Thermo Fisher) for 2 h and left to destain overnight in destaining solution (10% acetic acid–20% methanol). The destained gel pieces were incised, washed with high-performance liquid chromatography (HPLC)-grade water, and dehydrated with neat acetonitrile (ACN). Proteins were then reduced with 2 mM Tris(2-carboxyethyl)phosphine (TCEP) in 50 mM ammonium bicarbonate (AmBic) (NH_4_HCO_3_) for 1 h at 37°C and dehydrated with ACN. Reduced proteins were alkylated with 50 mM iodoacetamide (IAA)–50 mM AmBic for 20 min in the dark, with rotation. Gel pieces were then dehydrated for a second time with ACN to remove all reagents. Mass-spectrometry-grade trypsin (15 ng/μl) (Promega) was added to the samples and incubated for 30 min while on ice. After removal of excess trypsin, 25 mM AmBic was added to cover the gel pieces, which were incubated overnight at 37°C. Digested peptides were then extracted from the gel with a 50% ACN–0.1% trifluoroacetic acid solution. Samples were dried in a SpeedVac, dissolved in 15 μl formic acid (FA) (0.1%), and submitted for liquid chromatography-tandem mass spectrometry (LC-MS/MS) analysis.

In-gel-digested peptide samples were analyzed using an Orbitrap Fusion Lumos instrument coupled with an UltiMate 3000 HPLC system (Thermo Scientific). A total of 5 μl (500 ng) of each sample was loaded onto the trap column (Acclaim PepMap 100, 75 μm by 2 cm, with nanoViper; Thermo Scientific) using FA (0.1%) and resolved in a rapid-separation liquid chromatography (RSLC) column (Acclaim PepMap RSLC, 75 μm by 15 cm, with nanoViper; Thermo Scientific). Samples were eluted using a 90-min linear gradient of ACN (4% to 45%) in 0.1% FA. The parameters for all experiments were as follows: nanospray needle voltage in positive mode of 2,000 V, column flow rate of 0.3 μl/min, loading pump flow rate of 4 μl/min, and inject mode of μl PickUp. The Orbitrap scan mode was used for MS/MS, with a resolution of 120,000 and a scan range of *m/z* 375 to 1,500. Peptides were put into dynamic exclusion for 60 s after detection one time. The detector type for MS/MS was set to Orbitrap, with a resolution of 30,000, quadrupole isolation mode (isolation window of 0.7 Da), activation type of higher-energy collisional dissociation (HCD), HCD collision energy of 40%, and first mass of *m/z* 110. Stainless steel emitters were purchased from Thermo Fisher (outer diameter [OD] of 150 μm, internal diameter of 30 μm, and length of 40 mm, inserted into a 1/32 microsleeve for installation).

### Data analysis.

For cytokine and leukocyte infiltrate measurements, significant differences between experimental groups were determined using unpaired Student’s *t* test with SigmaStat (SPSS Science, Chicago, IL) at the 95% confidence interval. A *P* value of less than 0.05 was considered statistically significant.

For analysis of MS data, we filtered protein expression data that contained values for sterile and infected groups at days 1 and 5 postinfection. We applied principal-component analysis (PCA) to this multidimensional expression profile to reduce the dimension to fewer components. We used the “pheatmap” package in R (R Foundation for Statistical Computing, Vienna, Austria) to generate a heat map of expression for the highly variable proteins (49, 50). We used the permutation test for the difference between the means of two groups (sterile and infected) from each day and empirically calculated the *P* value with 1,000 random permutations of protein expression between the two groups. To adjust the false discovery rate due to multiple testing, we applied the Benjamini-Hochberg adjustment of the *P* value (51). Inbuilt functions such as p.adjust and external packages such as “VennDiagram” were used in R (R Foundation for Statistical Computing, Vienna, Austria) for multiple-testing correction and generating the Venn diagram (50, 52). We used the “PANTHER” functional enrichment tool to identify biological processes enriched in the differentially expressed proteins (53). Statistical significance of complement proteins was determined at day 1 and day 5 postinfection using the Wilcoxon signed-rank test.

## Supplementary Material

Supplemental file 1
