# Supplementary material for: Identification of Potential Cerebrospinal Fluid Biomarkers To Discriminate between Infection and Sterile Inflammation in a Rat Model of Staphylococcus epidermidis Catheter Infection
Source: Infect Immun. 2019 Aug 21;87(9):e00311-19. doi: 10.1128/IAI.00311-19 (PMC6704599; doi:10.1128/IAI.00311-19)
Supplement: Supplemental file 1 [file IAI.00311-19-s0001.pdf]

SUPPLEMENTARY INFORMATION

Table 1: Biological processes enriched at days 1 and 5 post-infection.

	PANTHER GO-Slim Biological Process	Total Reference Proteins	Number of Proteins	*p*-value
Day 1 Post-Infection	Negative regulation of endopeptidase activity (GO:0010951)	7	2	2.12E-05
	Negative regulation of peptidase activity (GO:0010466)	10	2	3.87E-05
	Negative regulation of hydrolase activity (GO:0051346)	18	2	1.11E-04
	Cellular protein metabolic process (GO:0044267)	130	7	6.78E-12
	Protein metabolic process (GO:0019538)	449	7	3.00E-08
	Macromolecule metabolic process (GO:0043170)	1583	7	1.20E-04
Day 5 Post-Infection	Positive regulation of biosynthetic process (GO:0009891)	4	2	4.54E-05
	Acylglycerol catabolic process (GO:0046464)	11	2	2.34E-04
	Lipoprotein metabolic process (GO:0042157)	11	2	2.34E-04
	Negative regulation of endopeptidase activity (GO:0010951)	7	4	2.71E-09
	Negative regulation of peptidase activity (GO:0010466)	10	4	8.19E-09
	Negative regulation of hydrolase activity (GO:0051346)	18	4	5.93E-08
	Negative regulation of catalytic activity (GO:0043086)	49	4	2.28E-06
	Proteolysis (GO:0006508)	137	4	1.10E-04
	Cellular protein metabolic process (GO:0044267)	130	12	1.00E-17
	Protein metabolic process (GO:0019538)	449	18	1.88E-20
	Macromolecule metabolic process (GO:0043170)	1583	19	3.01E-12
	Organic substance metabolic process (GO:0071704)	1998	19	1.68E-10
	Metabolic process (GO:0008152)	4840	21	1.27E-05
